# Supplementary material for: Rokubacteria: Genomic Giants among the Uncultured Bacterial Phyla
Source: Front Microbiol. 2017 Nov 28;8:2264. doi: 10.3389/fmicb.2017.02264 (PMC5712423; doi:10.3389/fmicb.2017.02264)
Supplement: Supplementary file 1 [file Presentation1.pdf]

**Supplemental Material for the Article Entitled ‘Rokubacteria: genomic giants among the uncultured Bacteria phyla’**

## Supplemental Section I: Predicted class-level metabolisms

All SAGs were examined in parallel for shared metabolic potential at the class-level, and the least contaminated metagenomic bins from OV-2 (bin 8) and *Tabebuia* (*T. heterophylla)* rhizosphere were used to supplement phylum-level metabolic analyses (Supplemental Figure 1).

In contrast to a prior report (Hug et al., 2016), we found that Rokubacteria SAG assemblies and metagenomic bins encode for a complete glycolytic pathway in addition to a complete TCA cycle and pentose phosphate pathway. Proteins predicted to be involved in aerobic respiration include components of the NADH dehydrogenase complex I, succinate dehydrogenase complex II and fumarate reductase, and the cytochrome *c* oxidase complex IV. Genes encoding for subunits of a putative F-type ATP synthase were identified, suggesting that these organisms have the capability for organic carbon acquisition and aerobic respiration. SAGs also contained genes for denitrification, including nitrate reductase and nitric acid reductase genes, although nitric oxide reductase and nitrous oxide reductase were not identified. Rokumicrobia SAGs encoded putative nitrite oxidoreductases, which are universally conserved in the Nitrospirae lineage. However, these proteins do not cluster with the experimentally verified uni-directional nitrite oxidoreductases of the Nitrospirae and Nitrospina (Supplemental Figure 7), and could be a bi-directional enzyme involved in nitrate reduction to nitrite instead (Sorokin et al., 2012). The Rokumicrobia SAG AD-967-E21 from the deep subsurface site in Finsch uniquely encodes nitrous oxide reductase (*nosZ*) in addition to nitrate reductase and nitrite reductase, and sulfur oxidation gene (*soxA*), which was also found in metagenomic bins from the Rifle site by a prior study (Hug et al., 2016). Coinciding this observation, Finsch mine

contained high levels of sulfate (93 mg/L), which may be the result of microbial sulfur oxidation (Supplemental Table 1). A sulfane dehydrogenase subunit gene (*soxC*) was detected in one Infratellusbacteria SAG; however, all other SAGs lacked sulfur oxidation pathway genes, indicating that they are unable to oxidize reduced sulfur sources for energy or that these genes remain undetected due to incomplete assemblies. Multiple SAGs in both classes also contained genes involved in arsenite/arsenate detoxification, including arsenite oxidase, arsenite reductase, and an arsenite-specific transporter. Overall, Rokubacteria can putatively utilize multiple election donors and acceptors.

Rokubacteria SAGs also contain genes involved in the fermentation of pyruvate to acetyl-CoA (*pdh*), acetyl-CoA to acetate (*acs*), acetate to acetaldehyde (ALDH), and acetaldehyde to ethanol (*adh*). Rokubacteria SAGs encode for the oxidative degradation of aromatic compounds, such as oxidative degradation of benzoyl-CoA and hydroxybenzoyl-CoA, and anaerobic enzymes benzoyl/hydroxybenzoyl-CoA reductase and phenylacetyl-CoA oxidoreductase, suggesting heterotrophic alternatives for energy production. Additionally, we found no evidence of methylotrophy (specifically methanol dehydrogenase) as reported in Butterfield et al. (Butterfield et al., 2016), though the Ribulose-P pathway to anabolize formaldehyde to fructose was identified.

Some Rokubacteria SAG assemblies contained pyruvate:ferredoxin oxidoreductase and acetyl-CoA reductase, two of the three diagnostic enzymes of the reverse TCA cycle (Figure 5 and Supplemental Table 5) (Hugler et al., 2005). However, ATP citrate lyase was not annotated. Further, Rokumicrobia SAGs contained carbon monoxide dehydrogenase, but lacked acetyl-CoA synthase, which are enzymes indicative of the Wood-Ljungdahl pathway (i.e. reverse acetyl-CoA

pathway). While incomplete, partial evidence for multiple autotrophic pathways in the same organism is interesting.

As mentioned in the main text, SAGs and metagenomic bins encode for multiple proteins involved in DNA packaging and super-coiling (Supplementary Table 5) (Dillon and Dorman, 2010), including topoisomerase (*top*), DNA gyrase (*gyr*), *parB* (chromosome partitioning protein), *hupB* (binding protein HU-beta), *ssb* (single-standed DNA binding protein), *cbpA*; curved DNA-binding protein and *smc* (chromosome segregation protein) (Supplemental Figure 1 and Supplemental Table 5). Rokumicrobia SAGs also contained integration host factors (IHF) which may associate with DNA in a non-specific manner contributing to bacterial chromatin organization, and *dps* (starvation-inducible DNA-binding protein) that organizes the nucleoid to protect it from potential threats (Lee et al., 2015). While many of the genes identified in Rokubacteria are present in well-studied bacteria, Rokubacteria are predicted to contain a variety of known DNA packaging mechanisms.

Rokumicrobia OV-2 assemblies encode complete pathways for dissimilatory nitrate reduction to ammonium (*narGHU*, *nirBD*) and assimilatory nitrate reduction (*narB*, *nirA*). Some additional interesting features of Rokubacteria SAGs include the ability to synthesize unusual lipids, including an unknown hopaniod derivative of bactriohopanetetrol and a putative phosphonolipid (located on the same contig in multiple SAGs). Numerous SAGs could putatively produce an unknown glycosylated pterin derivative encoded by a gene cluster including a protein tyrosine phosphatase (PTPS) enzyme. In addition, genomes derived from OV-2 sample encode a succinate dehydrogenase and fumarate reductase enzymes with high similarity to *Mycobacterium tuberculosis* (Hartman et al., 2014). These enzymes were not found in the genomes extracted from *Tabebuia* rhizosphere and Rifle sediment despite high degree of

completeness of these genomes, suggesting recent horizontal transfer or greater flexibility of respiratory chain metabolism than was previously annotated (Anantharaman et al., 2016).

It is important to note that all class-level metabolic predictions are inferred from genetically divergent and incomplete SAGs, and metagenomic bins containing genetically distinct individuals, though these inferences can provide an important first glimpse at the shared metabolic traits contained within these classes and phylum. We have confidence in predictions of metabolism that contain all, or nearly all, proteins in a given pathway, and are annotated with high confidence. However, many single proteins predicted to be present in uncultivated lineages are low confidence annotations, as reference genomes are evolutionarily distant, and metabolic predictions must always be interpreted with caution. Overall, both SAG and metagenomic bin content suggests that Rokubacteria have a generalist strategy for survival, using a mixotrophic metabolism for energy production.

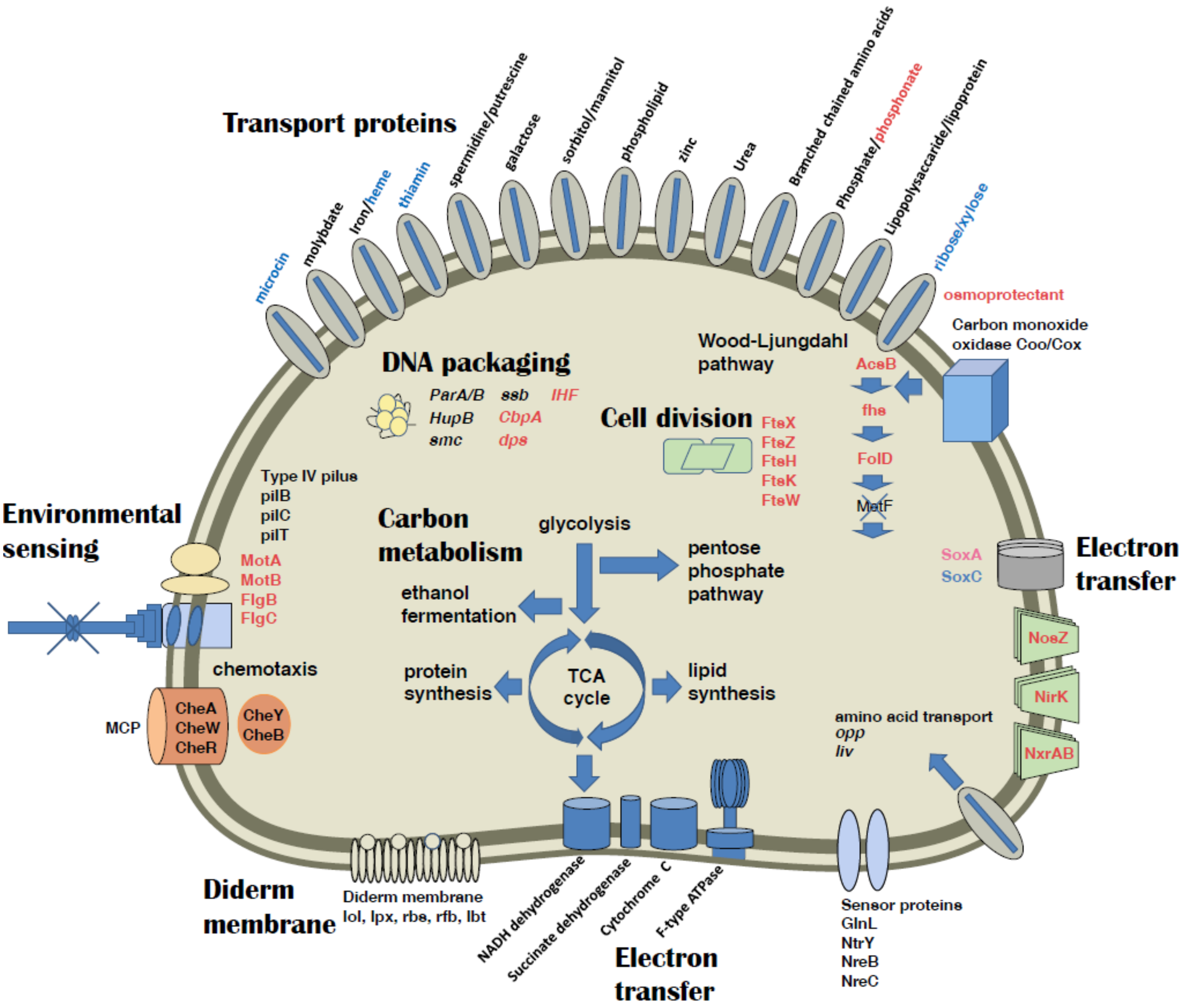


**Supplemental Figure 1.** Composite schematic diagram of predicted metabolic features in all Rokubacteria SAGs and metagenomics bins sequenced in this study. Black text indicates pathway/proteins found in both Rokubacteria classes. Blue text indicates pathways/proteins detected only in Infratellusbacteria. Red text indicates pathways/proteins found only in Rokumicrobia. Pink text indicates genes found in a single SAG from the Finsch mine (AD-967-E21). X represents a missing protein or pathway. Also see Supplementary Table 5.

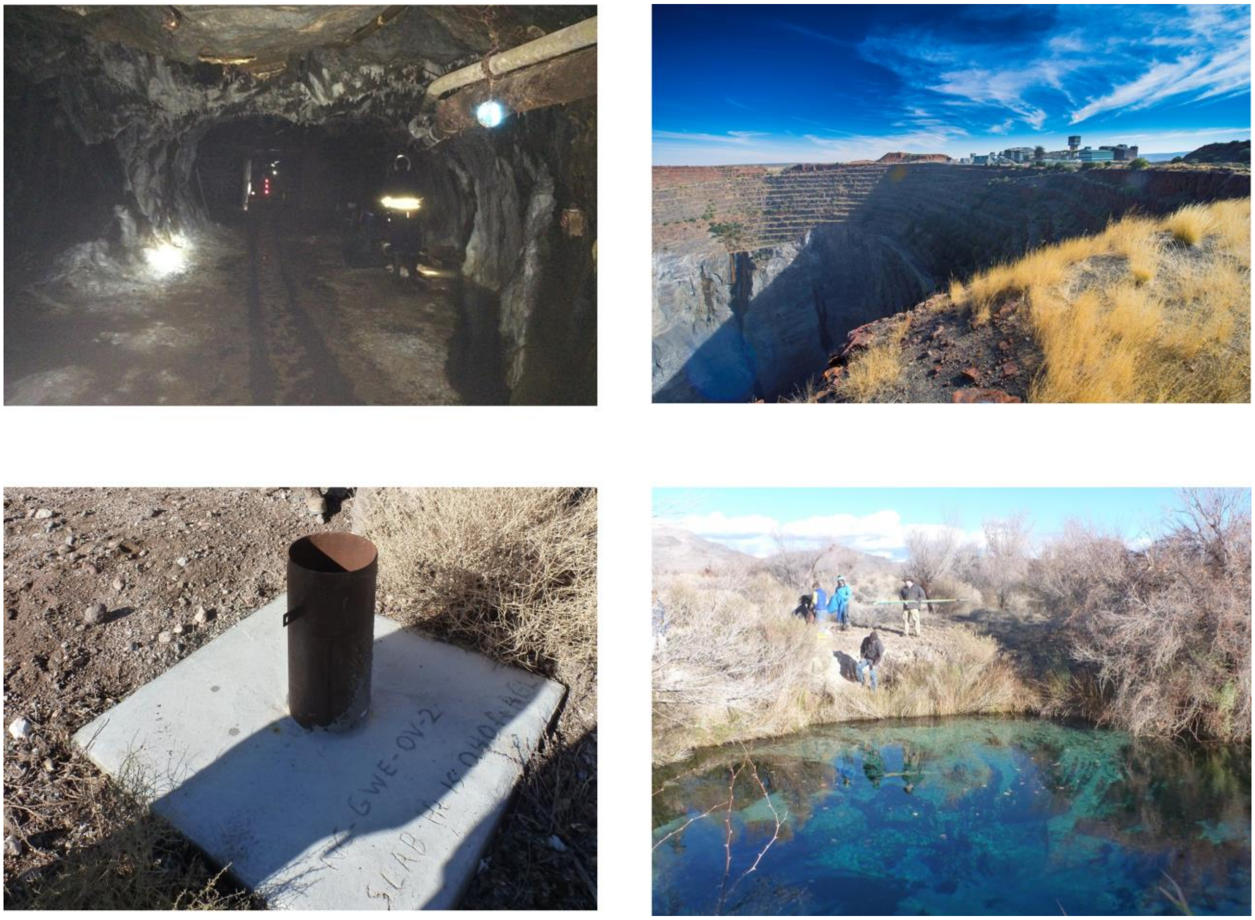


**Supplemental Figure 2.** Sample sites for SAG collection. Sanford Underground Research Facility (SURF; top left); Finsch gold mine in South Africa (top right); Nevada Nye County Groundwater Evaluation well Oasis Valley 2 (bottom left); Crystal Spring in Ash Meadows Nevada (bottom right).

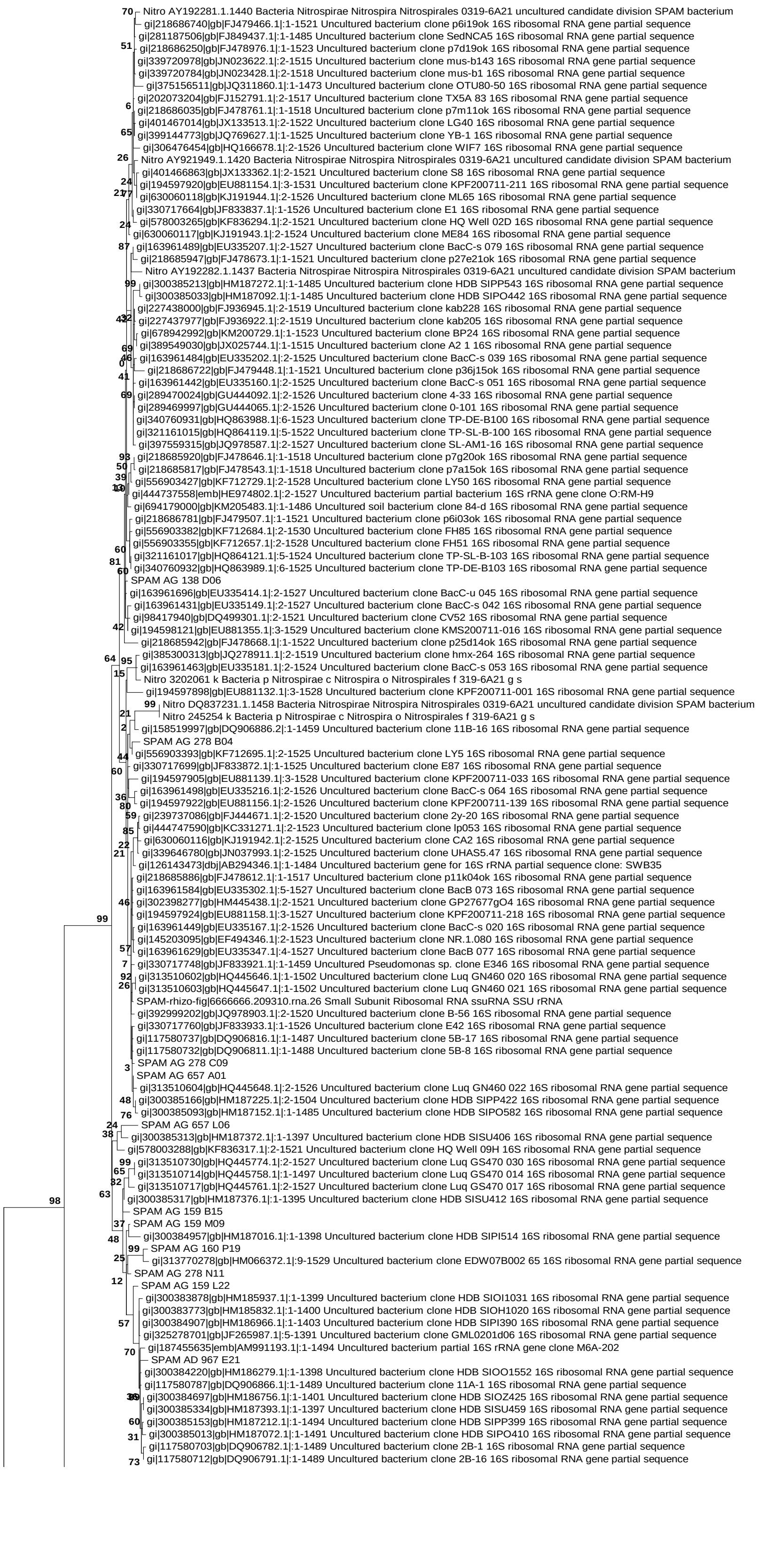

Nitro AY192281.1.1440 Bacteria Nitrospirae Nitrospira Nitrospirales 0319-6A21 uncultured candidate division SPAM bacterium
gi|218686740|gb|FJ479466.1|:1-1521 Uncultured bacterium clone p6i19ok 16S ribosomal RNA gene partial sequence
gi|281187506|gb|FJ849437.1|:1-1485 Uncultured bacterium clone SedNCA5 16S ribosomal RNA gene partial sequence
gi|218686250|gb|FJ478976.1|:1-1523 Uncultured bacterium clone p7d19ok 16S ribosomal RNA gene partial sequence
gi|339720978|gb|JN023622.1|:2-1515 Uncultured bacterium clone mus-b143 16S ribosomal RNA gene partial sequence
gi|339720784|gb|JN023428.1|:2-1518 Uncultured bacterium clone mus-b1 16S ribosomal RNA gene partial sequence
gi|375156511|gb|JQ311860.1|:1-1473 Uncultured bacterium clone OTU80-50 16S ribosomal RNA gene partial sequence
gi|202073204|gb|FJ152791.1|:2-1517 Uncultured bacterium clone TX5A 83 16S ribosomal RNA gene partial sequence
gi|218686035|gb|FJ478761.1|:1-1518 Uncultured bacterium clone p7m11ok 16S ribosomal RNA gene partial sequence
gi|401467014|gb|JX133513.1|:2-1522 Uncultured bacterium clone LG40 16S ribosomal RNA gene partial sequence
gi|399144773|gb|JQ769627.1|:1-1525 Uncultured bacterium clone YB-1 16S ribosomal RNA gene partial sequence
gi|306476454|gb|HQ166678.1|:2-1526 Uncultured bacterium clone WIF7 16S ribosomal RNA gene partial sequence
Nitro AY921949.1.1420 Bacteria Nitrospirae Nitrospira Nitrospirales 0319-6A21 uncultured candidate division SPAM bacterium
gi|401466863|gb|JX133362.1|:2-1521 Uncultured bacterium clone S8 16S ribosomal RNA gene partial sequence
gi|194597920|gb|EU881154.1|:3-1531 Uncultured bacterium clone KPF200711-211 16S ribosomal RNA gene partial sequence
gi|630060118|gb|KJ191944.1|:2-1526 Uncultured bacterium clone ML65 16S ribosomal RNA gene partial sequence
gi|330717664|gb|JF833837.1|:1-1526 Uncultured bacterium clone E1 16S ribosomal RNA gene partial sequence
gi|578003265|gb|KF836294.1|:2-1521 Uncultured bacterium clone HQ Well 02D 16S ribosomal RNA gene partial sequence
gi|630060117|gb|KJ191943.1|:2-1524 Uncultured bacterium clone ME84 16S ribosomal RNA gene partial sequence
gi|163961489|gb|EU335207.1|:2-1527 Uncultured bacterium clone BacC-s 079 16S ribosomal RNA gene partial sequence
gi|218685947|gb|FJ478673.1|:1-1521 Uncultured bacterium clone p27e21ok 16S ribosomal RNA gene partial sequence
Nitro AY192282.1.1437 Bacteria Nitrospirae Nitrospira Nitrospirales 0319-6A21 uncultured candidate division SPAM bacterium
gi|300385213|gb|HM187272.1|:1-1485 Uncultured bacterium clone HDB SIPP543 16S ribosomal RNA gene partial sequence
gi|300385033|gb|HM187092.1|:1-1485 Uncultured bacterium clone HDB SIPO442 16S ribosomal RNA gene partial sequence
gi|227438000|gb|FJ936945.1|:2-1519 Uncultured bacterium clone kab228 16S ribosomal RNA gene partial sequence
gi|227437977|gb|FJ936922.1|:2-1519 Uncultured bacterium clone kab205 16S ribosomal RNA gene partial sequence
gi|678942992|gb|KM200729.1|:1-1523 Uncultured bacterium clone BP24 16S ribosomal RNA gene partial sequence
gi|389549030|gb|JX025744.1|:1-1515 Uncultured bacterium clone A2 1 16S ribosomal RNA gene partial sequence
gi|163961484|gb|EU335202.1|:2-1525 Uncultured bacterium clone BacC-s 039 16S ribosomal RNA gene partial sequence
gi|218686722|gb|FJ479448.1|:1-1521 Uncultured bacterium clone p36j15ok 16S ribosomal RNA gene partial sequence
gi|163961442|gb|EU335160.1|:2-1525 Uncultured bacterium clone BacC-s 051 16S ribosomal RNA gene partial sequence
gi|289470024|gb|GU444092.1|:2-1526 Uncultured bacterium clone 4-33 16S ribosomal RNA gene partial sequence
gi|289469997|gb|GU444065.1|:2-1526 Uncultured bacterium clone 0-101 16S ribosomal RNA gene partial sequence
gi|340760931|gb|HQ863988.1|:6-1523 Uncultured bacterium clone TP-DE-B100 16S ribosomal RNA gene partial sequence
gi|321161015|gb|HQ864119.1|:5-1522 Uncultured bacterium clone TP-SL-B-100 16S ribosomal RNA gene partial sequence
gi|397559315|gb|JQ978587.1|:2-1527 Uncultured bacterium clone SL-AM1-16 16S ribosomal RNA gene partial sequence
gi|218685920|gb|FJ478646.1|:1-1518 Uncultured bacterium clone p7g20ok 16S ribosomal RNA gene partial sequence
gi|218685817|gb|FJ478543.1|:1-1518 Uncultured bacterium clone p7a15ok 16S ribosomal RNA gene partial sequence
gi|556903427|gb|KF712729.1|:2-1528 Uncultured bacterium clone LY50 16S ribosomal RNA gene partial sequence
gi|444737558|emb|HE974802.1|:2-1527 Uncultured bacterium partial bacterium 16S rRNA gene clone O:RM-H9
gi|694179000|gb|KM205483.1|:1-1486 Uncultured soil bacterium clone 84-d 16S ribosomal RNA gene partial sequence
gi|218686781|gb|FJ479507.1|:1-1521 Uncultured bacterium clone p6i03ok 16S ribosomal RNA gene partial sequence
gi|556903382|gb|KF712684.1|:2-1530 Uncultured bacterium clone FH85 16S ribosomal RNA gene partial sequence
gi|556903355|gb|KF712657.1|:2-1528 Uncultured bacterium clone FH51 16S ribosomal RNA gene partial sequence
gi|321161017|gb|HQ864121.1|:5-1524 Uncultured bacterium clone TP-SL-B-103 16S ribosomal RNA gene partial sequence
gi|340760932|gb|HQ863989.1|:6-1525 Uncultured bacterium clone TP-DE-B103 16S ribosomal RNA gene partial sequence
SPAM AG 138 D06
gi|163961696|gb|EU335414.1|:2-1527 Uncultured bacterium clone BacC-u 045 16S ribosomal RNA gene partial sequence
gi|163961431|gb|EU335149.1|:2-1527 Uncultured bacterium clone BacC-s 042 16S ribosomal RNA gene partial sequence
gi|98417940|gb|DQ499301.1|:2-1521 Uncultured bacterium clone CV52 16S ribosomal RNA gene partial sequence
gi|194598121|gb|EU881355.1|:3-1529 Uncultured bacterium clone KMS200711-016 16S ribosomal RNA gene partial sequence
gi|218685942|gb|FJ478668.1|:1-1522 Uncultured bacterium clone p25d14ok 16S ribosomal RNA gene partial sequence
gi|385300313|gb|JQ278911.1|:2-1519 Uncultured bacterium clone hmx-264 16S ribosomal RNA gene partial sequence
gi|163961463|gb|EU335181.1|:2-1524 Uncultured bacterium clone BacC-s 053 16S ribosomal RNA gene partial sequence
Nitro 3202061 k Bacteria p Nitrospirae c Nitrospira o Nitrospirales f 319-6A21 g s
gi|194597898|gb|EU881132.1|:3-1528 Uncultured bacterium clone KPF200711-001 16S ribosomal RNA gene partial sequence
Nitro DQ837231.1.1458 Bacteria Nitrospirae Nitrospira Nitrospirales 0319-6A21 uncultured candidate division SPAM bacterium
Nitro 245254 k Bacteria p Nitrospirae c Nitrospira o Nitrospirales f 319-6A21 g s
gi|158519997|gb|DQ906886.2|:1-1459 Uncultured bacterium clone 11B-16 16S ribosomal RNA gene partial sequence
SPAM AG 278 B04
gi|556903393|gb|KF712695.1|:2-1525 Uncultured bacterium clone LY5 16S ribosomal RNA gene partial sequence
gi|330717699|gb|JF833872.1|:1-1525 Uncultured bacterium clone E87 16S ribosomal RNA gene partial sequence
gi|194597905|gb|EU881139.1|:3-1528 Uncultured bacterium clone KPF200711-033 16S ribosomal RNA gene partial sequence
gi|163961498|gb|EU335216.1|:2-1526 Uncultured bacterium clone BacC-s 064 16S ribosomal RNA gene partial sequence
gi|194597922|gb|EU881156.1|:2-1526 Uncultured bacterium clone KPF200711-139 16S ribosomal RNA gene partial sequence
gi|239737086|gb|FJ444671.1|:2-1520 Uncultured bacterium clone 2y-20 16S ribosomal RNA gene partial sequence
gi|444747590|gb|KC331271.1|:2-1523 Uncultured bacterium clone lp053 16S ribosomal RNA gene partial sequence
gi|630060116|gb|KJ191942.1|:2-1525 Uncultured bacterium clone CA2 16S ribosomal RNA gene partial sequence
gi|339646780|gb|JN037993.1|:2-1525 Uncultured bacterium clone UHAS5.47 16S ribosomal RNA gene partial sequence
gi|126143473|dbj|AB294346.1|:1-1484 Uncultured bacterium gene for 16S rRNA partial sequence clone: SWB35
gi|218685886|gb|FJ478612.1|:1-1517 Uncultured bacterium clone p11k04ok 16S ribosomal RNA gene partial sequence
gi|163961584|gb|EU335302.1|:5-1527 Uncultured bacterium clone BacB 073 16S ribosomal RNA gene partial sequence
gi|302398277|gb|HM445438.1|:2-1521 Uncultured bacterium clone GP27677gO4 16S ribosomal RNA gene partial sequence
gi|194597924|gb|EU881158.1|:3-1527 Uncultured bacterium clone KPF200711-218 16S ribosomal RNA gene partial sequence
gi|163961449|gb|EU335167.1|:2-1526 Uncultured bacterium clone BacC-s 020 16S ribosomal RNA gene partial sequence
gi|145203095|gb|EF494346.1|:2-1523 Uncultured bacterium clone NR.1.080 16S ribosomal RNA gene partial sequence
gi|163961629|gb|EU335347.1|:4-1527 Uncultured bacterium clone BacB 077 16S ribosomal RNA gene partial sequence
gi|330717748|gb|JF833921.1|:1-1459 Uncultured Pseudomonas sp. clone E346 16S ribosomal RNA gene partial sequence
gi|313510602|gb|HQ445646.1|:1-1502 Uncultured bacterium clone Luq GN460 020 16S ribosomal RNA gene partial sequence
gi|313510603|gb|HQ445647.1|:1-1502 Uncultured bacterium clone Luq GN460 021 16S ribosomal RNA gene partial sequence
SPAM-rhizo-fig|6666666.209310.rna.26 Small Subunit Ribosomal RNA ssuRNA SSU rRNA
gi|392999202|gb|JQ978903.1|:2-1520 Uncultured bacterium clone B-56 16S ribosomal RNA gene partial sequence
gi|330717760|gb|JF833933.1|:1-1526 Uncultured bacterium clone E42 16S ribosomal RNA gene partial sequence
gi|117580737|gb|DQ906816.1|:1-1487 Uncultured bacterium clone 5B-17 16S ribosomal RNA gene partial sequence
gi|117580732|gb|DQ906811.1|:1-1488 Uncultured bacterium clone 5B-8 16S ribosomal RNA gene partial sequence
SPAM AG 278 C09
SPAM AG 657 A01
gi|313510604|gb|HQ445648.1|:2-1526 Uncultured bacterium clone Luq GN460 022 16S ribosomal RNA gene partial sequence
gi|300385166|gb|HM187225.1|:2-1504 Uncultured bacterium clone HDB SIPP422 16S ribosomal RNA gene partial sequence
gi|300385093|gb|HM187152.1|:1-1485 Uncultured bacterium clone HDB SIPO582 16S ribosomal RNA gene partial sequence
SPAM AG 657 L06
gi|300385313|gb|HM187372.1|:1-1397 Uncultured bacterium clone HDB SISU406 16S ribosomal RNA gene partial sequence
gi|578003288|gb|KF836317.1|:2-1521 Uncultured bacterium clone HQ Well 09H 16S ribosomal RNA gene partial sequence
gi|313510730|gb|HQ445774.1|:2-1527 Uncultured bacterium clone Luq GS470 030 16S ribosomal RNA gene partial sequence
gi|313510714|gb|HQ445758.1|:1-1497 Uncultured bacterium clone Luq GS470 014 16S ribosomal RNA gene partial sequence
gi|313510717|gb|HQ445761.1|:2-1527 Uncultured bacterium clone Luq GS470 017 16S ribosomal RNA gene partial sequence
gi|300385317|gb|HM187376.1|:1-1395 Uncultured bacterium clone HDB SISU412 16S ribosomal RNA gene partial sequence
SPAM AG 159 B15
SPAM AG 159 M09
gi|300384957|gb|HM187016.1|:1-1398 Uncultured bacterium clone HDB SIPI514 16S ribosomal RNA gene partial sequence
SPAM AG 160 P19
gi|313770278|gb|HM066372.1|:9-1529 Uncultured bacterium clone EDW07B002 65 16S ribosomal RNA gene partial sequence
SPAM AG 278 N11
SPAM AG 159 L22
gi|300383878|gb|HM185937.1|:1-1399 Uncultured bacterium clone HDB SIOI1031 16S ribosomal RNA gene partial sequence
gi|300383773|gb|HM185832.1|:1-1400 Uncultured bacterium clone HDB SIOH1020 16S ribosomal RNA gene partial sequence
gi|300384907|gb|HM186966.1|:1-1403 Uncultured bacterium clone HDB SIPI390 16S ribosomal RNA gene partial sequence
gi|325278701|gb|JF265987.1|:5-1391 Uncultured bacterium clone GML0201d06 16S ribosomal RNA gene partial sequence
gi|187455635|emb|AM991193.1|:1-1494 Uncultured bacterium partial 16S rRNA gene clone M6A-202
SPAM AD 967 E21
gi|300384220|gb|HM186279.1|:1-1398 Uncultured bacterium clone HDB SIOO1552 16S ribosomal RNA gene partial sequence
gi|117580787|gb|DQ906866.1|:1-1489 Uncultured bacterium clone 11A-1 16S ribosomal RNA gene partial sequence
gi|300384697|gb|HM186756.1|:1-1401 Uncultured bacterium clone HDB SIOZ425 16S ribosomal RNA gene partial sequence
gi|300385334|gb|HM187393.1|:1-1397 Uncultured bacterium clone HDB SISU459 16S ribosomal RNA gene partial sequence
gi|300385153|gb|HM187212.1|:1-1494 Uncultured bacterium clone HDB SIPP399 16S ribosomal RNA gene partial sequence
gi|300385013|gb|HM187072.1|:1-1491 Uncultured bacterium clone HDB SIPO410 16S ribosomal RNA gene partial sequence
gi|117580703|gb|DQ906782.1|:1-1489 Uncultured bacterium clone 2B-1 16S ribosomal RNA gene partial sequence
gi|117580712|gb|DQ906791.1|:1-1489 Uncultured bacterium clone 2B-16 16S ribosomal RNA gene partial sequence

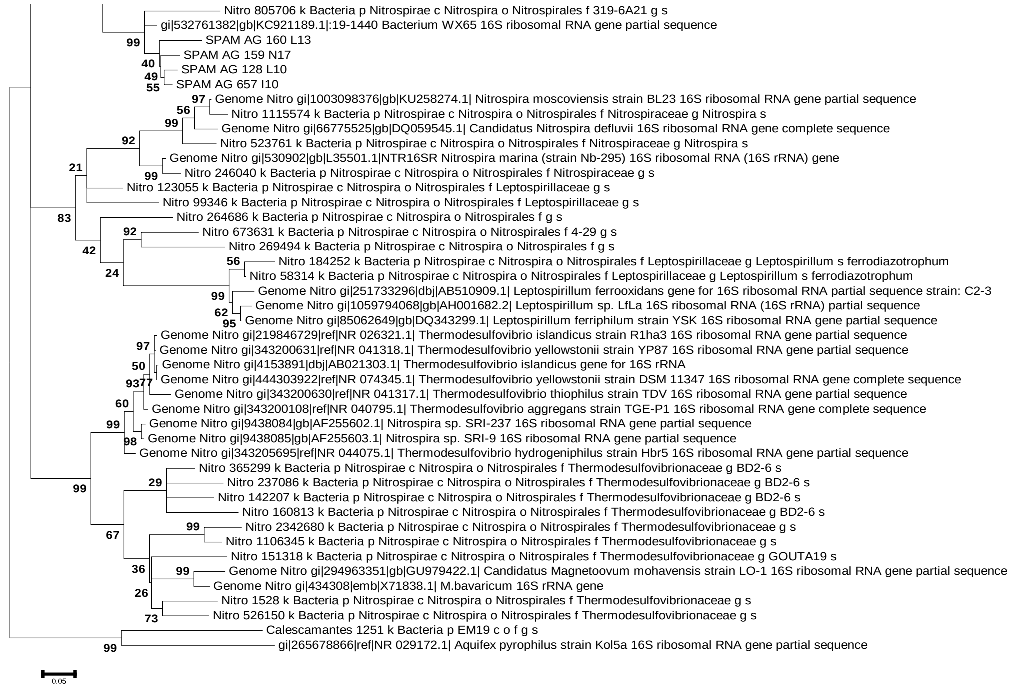


**Supplemental Figure 3.** 16S rRNA gene phylogeny of sequences in GenBank that were ≥ 85% identity to Rokubacteria SAG 16S rRNA gene sequences and representative Nitrospirae sequences from all classified genera, including sequence identifiers (also see Figure 1).

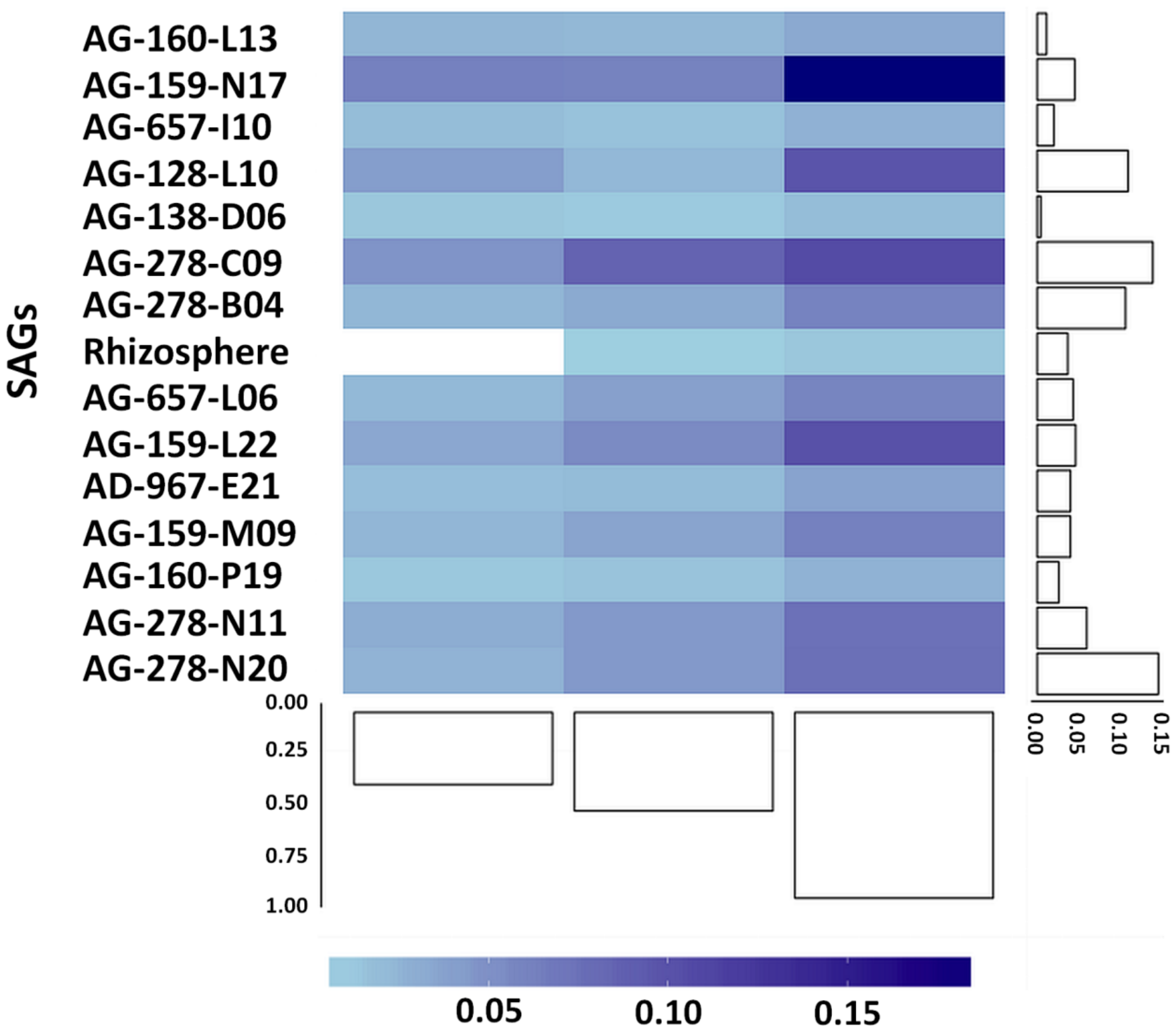


**Supplemental Figure 4.** Read mapping of OV-2 metagenomic reads to Rokubacteria SAGs sequenced from OV-2 and the *Tabebuia* rhizosphere metagenomic assembly. Samples from left to right are P1, P2, and P3 (see methods). Darker colors represent a higher fraction of reads mapped to individual SAGs (scale bar on bottom). Bars on right axis indicate the fraction of reads mapped to each individual SAG in all samples; bars on the bottom axis represent percent read abundance in the total sample.

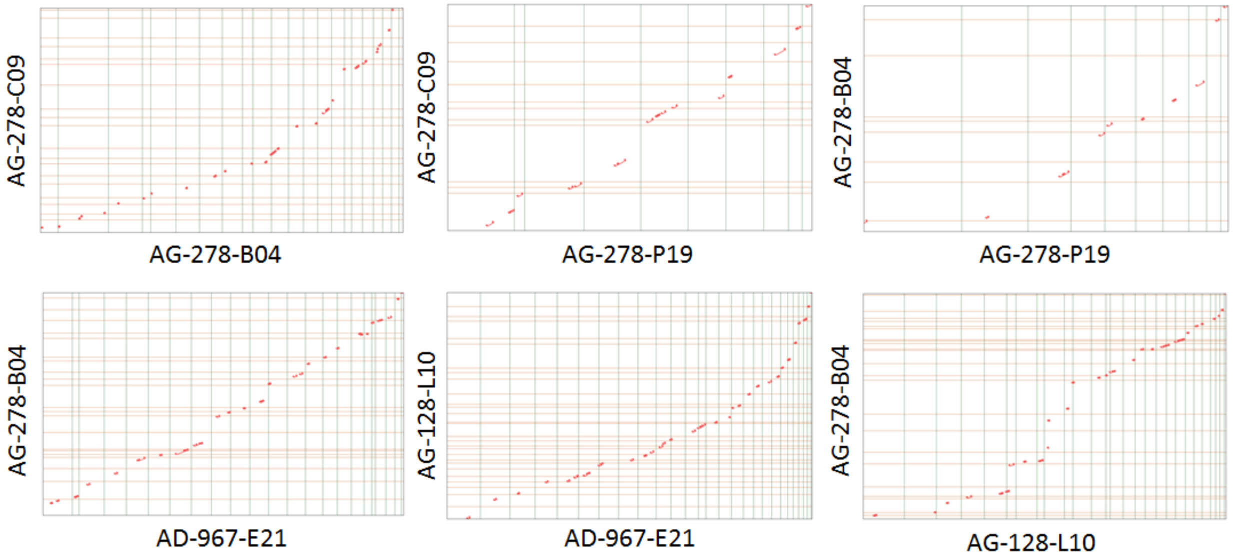


**Supplemental Figure 5.** Synteny plots for artificially aligned contigs (nucleotide) for 6 of the most complete Rokubacteria single simplified genomes (SAGs) analyzed at the Joint Genome Center's Integrated Microbial Genomes website. Red dots indicate genes with shared locations between artificially aligned contigs, represented by vertical lines.

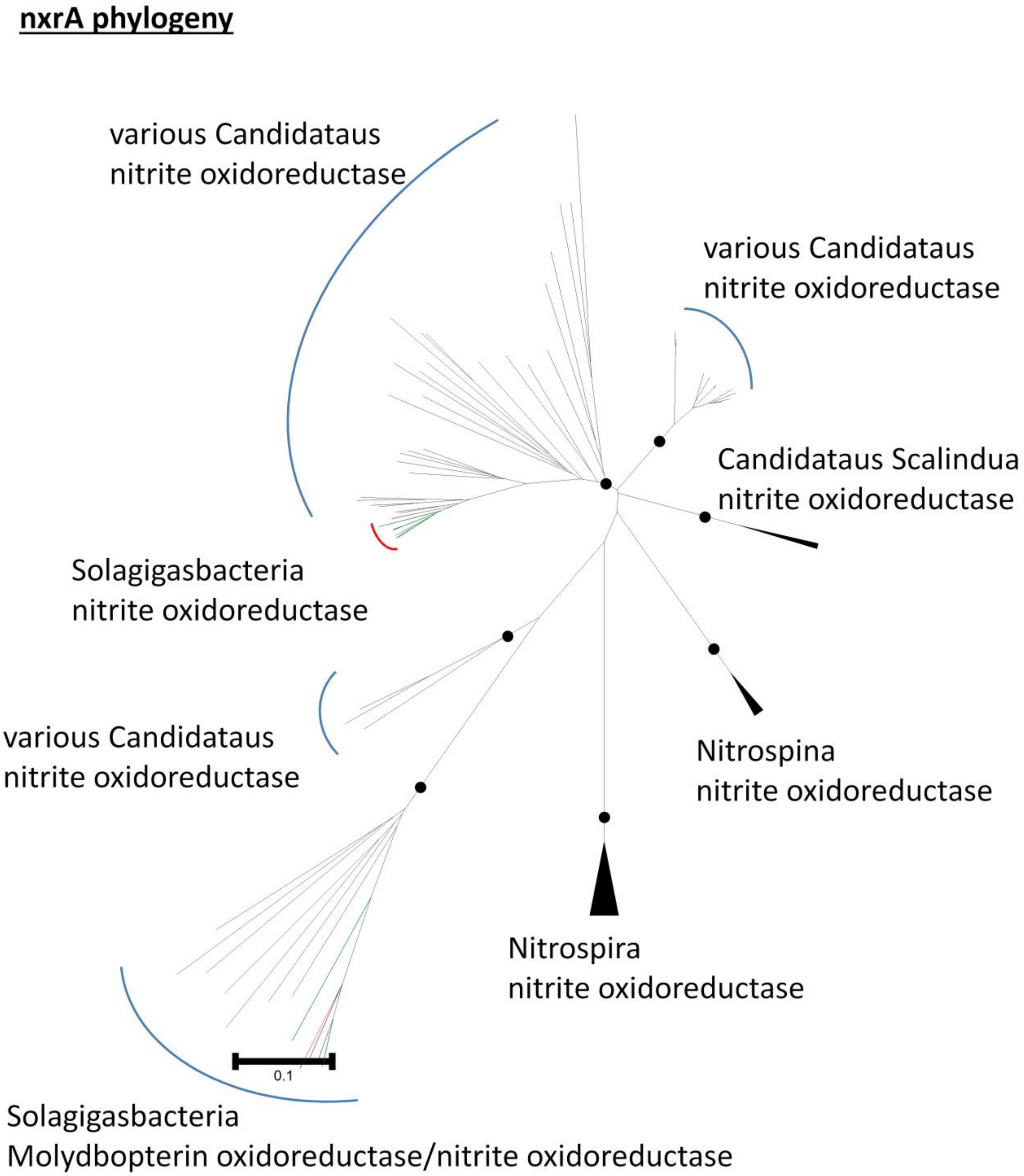


**Supplemental Figure 6.** Neighbor-joining phylogeny (100X boot-strapped) of the top 100 non-environmental NxrA sequences in Genbank identified by BLASTp alignment to annotated NxrA Rokubacteria proteins. Black circles indicate boot strap values > 90%. Red lines indicate Rokumicrobia SAG sequences, and green lines represent previously annotated Rokumicrobia metagenomic bin sequences (Anantharaman et al., 2016). Tree was rooted with known Nitrospirae NxrA sequences.

**Supplemental Table 1.** Geochemical data and locations of sites that samples for single cell genomics were collected for sorting and sequencing.

| | **Crystal Spring** | **OV-2 P3** | **Finsch** | **SURF** |
|---|---|---|---|---|
| Latitude | 36.4202 N | 36.960 N | -28.378 S | 44.35 N |
| Longitude | -116.3233 W | -116.720 W | 23.446 E | -103.756 W |
| Water Temp (°C) | 28.49 | 25.59 | 28.6 | 9.4 |
| Water pH | 7.26 | 7.44 | 6.8 | 7.71 |
| Conductivity (µS/cm) | 782 | 1363 | 470 | 627 |
| Dissolved oxygen (mg/L) | 3.7 | 2.54 | 0.025 | 8 |
| Sulfate (mg/L) | 89 | 181 | 93 | N/A |
| Depth (meters) | 0 | 9.1 – 27.4 m | 857 | 300 |

**Supplemental Table 2.** Pairwise 16S rRNA gene nucleotide identity for all Rokubacteria single simplified genomes (SAGs).

| | AG-278-N11 | AG-159-M09 | AG-159-L22 | AG-159-B15 | AG-278-A09 | AG-278-N20 | AD-967-E21 | AG-657-L06 | AG-160-P19 | AG-657-A01 | AG-138-D06 | AG-278-C09 | AG-278-B04 | AG-159-N17 | AG-128-L10 | AG-657-I10 | AG-160-L13 |
|---|---|---|---|---|---|---|---|---|---|---|---|---|---|---|---|---|---|
| AG-278-N11 | 100 | 97.56 | 96.17 | 95.01 | 96.75 | 97.1 | 94.41 | 94.05 | 93.87 | 93.72 | 92.94 | 91.45 | 91.25 | 87.43 | 86.41 | 85.9 | 85.2 |
| AG-159-M09 | 97.56 | 100 | 96.59 | 96.25 | 97.73 | 96.36 | 93.48 | 94.29 | 94.04 | 94.42 | 93.34 | 92.4 | 90.93 | 87.78 | 86.68 | 86.29 | 86.18 |
| AG-159-L22 | 96.17 | 96.59 | 100 | 95.58 | 97.09 | 96.08 | 93.76 | 93.93 | 93.81 | 93.66 | 92.67 | 91.75 | 89.82 | 87.84 | 86.74 | 86.29 | 85.57 |
| AG-159-B15 | 95.58 | 96.25 | 95.01 | 100 | 96.04 | 95.24 | 92.64 | 93.03 | 92.19 | 94.04 | 91.44 | 91.95 | 90.71 | 86.65 | 86.88 | 85.42 | 87.67 |
| AG-278-A09 | 96.75 | 97.73 | 97.09 | 96.04 | 100 | 96.8 | 92.53 | 94.41 | 92.89 | 94.12 | 93.34 | 92.41 | 90.81 | 88.25 | 86.39 | 86.27 | 85.91 |
| AG-278-N20 | 97.1 | 96.36 | 96.08 | 95.24 | 96.8 | 100 | 94.16 | 93.2 | 93.35 | 94.14 | 92.78 | 92.17 | 91.21 | 87.71 | 86.84 | 86.27 | 85.91 |
| AD-967-E21 | 94.41 | 93.48 | 93.76 | 91.44 | 92.53 | 94.16 | 100 | 92.58 | 92.24 | 93.3 | 91.28 | 90.96 | 89.93 | 85.66 | 85 | 84.9 | 83.92 |
| AG-657-L06 | 94.05 | 94.29 | 93.93 | 94.04 | 94.41 | 93.2 | 92.58 | 100 | 92.25 | 92.52 | 92.95 | 92.59 | 91.25 | 87.2 | 86.46 | 86.21 | 86.39 |
| AG-160-P19 | 93.87 | 94.04 | 93.81 | 92.19 | 92.89 | 93.35 | 92.24 | 92.25 | 100 | 92.56 | 90.95 | 91.1 | 89.88 | 86.61 | 86.61 | 85.38 | 86.98 |
| AG-657-A01 | 93.72 | 94.42 | 93.66 | 93.03 | 94.12 | 94.14 | 93.3 | 92.52 | 92.56 | 100 | 95.26 | 96.8 | 96.07 | 86.74 | 86.82 | 86.64 | 85.63 |
| AG-138-D06 | 92.94 | 93.34 | 92.67 | 92.64 | 93.34 | 92.78 | 91.28 | 92.95 | 90.96 | 95.26 | 100 | 94.72 | 91.99 | 86.67 | 86.44 | 85.66 | 85.31 |
| AG-278-C09 | 91.47 | 92.42 | 91.76 | 91.94 | 92.41 | 92.17 | 90.96 | 92.59 | 91.1 | 96.8 | 94.72 | 100 | 95.77 | 87.17 | 85.5 | 85.4 | 84.39 |
| AG-278-B04 | 91.26 | 90.93 | 89.84 | 90.71 | 90.81 | 91.21 | 89.93 | 91.25 | 89.88 | 96.07 | 91.99 | 95.77 | 100 | 85.54 | 85.2 | 84.64 | 82.81 |
| AG-159-N17 | 87.43 | 87.78 | 87.84 | 87.67 | 88.25 | 87.71 | 85.66 | 87.2 | 86.61 | 86.7 | 86.67 | 87.17 | 85.54 | 100 | 93.46 | 94.34 | 92.32 |
| AG-128-L10 | 86.37 | 86.68 | 86.74 | 86.88 | 86.39 | 86.84 | 84.96 | 86.46 | 86.61 | 86.82 | 86.44 | 85.5 | 85.14 | 93.46 | 100 | 96.16 | 90.78 |
| AG-657-I10 | 86.37 | 86.68 | 86.74 | 86.88 | 86.27 | 86.27 | 84.96 | 86.46 | 86.61 | 86.82 | 86.44 | 85.5 | 85.14 | 93.46 | 96.16 | 100 | 90.78 |
| AG-160-L13 | 85.2 | 86.18 | 85.57 | 85.42 | 85.91 | 85.91 | 83.92 | 86.39 | 86.98 | 85.63 | 85.29 | 84.39 | 82.81 | 92.32 | 90.78 | 90.85 | 100 |

**Supplemental Table 3.** Riffle Rokumicrobia metagenome bin assembly statistics and predicted genome completeness from (Anantharaman et al., 2016). Genome completeness and contamination were estimated using checkM (Parks et al., 2015).

| Rokubacteria SAGs | Class | Site | Assembly (Mbps) | GC % | Genome completeness, % | Contamination, % |
|---|---|---|---|---|---|---|
| RIFCSPLOWO2_02_73 | Rokubacteria | Riffle | 4.05 | 73.4 | 87.6 | 4.81 |
| RIFCSPLOWO2_12_73 | Rokubacteria | Riffle | 4.04 | 73.4 | 87.0 | 2.67 |
| RIFCSPLOWO2_02_72 | Rokubacteria | Riffle | 2.39 | 71.8 | 64.1 | 1.71 |
| GWA2_73_35 | Rokubacteria | Riffle | 3.54 | 72.8 | 71.2 | 2.67 |
| RIFCSPHIGHO2_02_73 | Rokubacteria | Riffle | 4.36 | 72.9 | 84.3 | 5.77 |
| GWC2_70_24 | Rokubacteria | Riffle | 2.94 | 70.2 | 75.7 | 1.82 |
| GWA2_70_23 | Rokubacteria | Riffle | 4.66 | 70.0 | 84.2 | 2.96 |
| RIFCSPHIGHO2_12_73 | Rokubacteria | Riffle | 3.62 | 72.8 | 68.3 | 2.67 |
| RIFCSPLOWO2_12_69 | Rokubacteria | Riffle | 2.81 | 68.1 | 70.2 | 14.22 |
| RIFCSPLOWO2_12_71 | Rokubacteria | Riffle | 3.44 | 71.4 | 68.3 | 4.73 |
| RBG_16_73_20 | Rokubacteria | Riffle | 3.76 | 71.9 | 67.6 | 2.04 |
| RIFCSPLOWO2_02_68 | Rokubacteria | Riffle | 3.09 | 67.1 | 79.5 | 2.69 |
| RIFCSPLOWO2_02_71 | Rokubacteria | Riffle | 1.04 | 68.3 | 31.3 | 0.38 |
| RIFCSPLOWO2_12_71 | Rokubacteria | Riffle | 3.48 | 70.8 | 63.7 | 3.44 |
| GWC2_70_16 | Rokubacteria | Riffle | 4.70 | 70.9 | 77.9 | 1.82 |
| GWF2_70_14 | Rokubacteria | Riffle | 3.32 | 69.3 | 66.5 | 2.14 |
| RIFCSPHIGHO2_02_69 | Rokubacteria | Riffle | 2.58 | 64.2 | 54.4 | 5.66 |

**Supplemental Table 4.** Rokubacteria mobile elements and viral signatures.

| Rokubacteria SAGs | Class | Site | Mobile element count | Phage genes | CRISPRs |
|---|---|---|---|---|---|
| ***AD-967-E21** | Rokumicrobia | Finsch | 60 (3.8%) | 14 | 5 |
| ***AG-128-L10** | Infratellusbacteria | CS[1] | 38 (2.2%) | 12 | 2 |
| AG-138-D06 | Rokumicrobia | SURF[2] | 7 (2.6%) | 3 | 1 |
| AG-657-A01 | Rokumicrobia | OV-2 | 14 (3.8%) | 2 | 0 |
| ***AG-657-I10** | Infratellusbacteria | OV-2 | 21 (2.2%) | 7 | 2 |
| ***AG-657-L06** | Rokumicrobia | OV-2 | 26 (2.5%) | 6 | 0 |
| AG-159-B15 | Rokumicrobia | OV-2 | 3 (5.5%) | 0 | 0 |
| AG-159-G23 | Rokumicrobia | OV-2 | 23 (5.3%) | 3 | 0 |
| ***AG-159-L22** | Rokumicrobia | OV-2 | 21 (3.1%) | 4 | 2 |
| ***AG-159-M09** | Rokumicrobia | OV-2 | 21 (2.2%) | 7 | 1 |
| AG-159-N17 | Infratellusbacteria | OV-2 | 28 (2.46%) | 6 | 0 |
| AG-159-P01 | Rokumicrobia | OV-2 | 3 (0.4%) | 0 | 0 |
| AG-160-L13 | Infratellusbacteria | OV-2 | 10 (2.4%) | 1 | 1 |
| ***AG-160-P19** | Rokumicrobia | OV-2 | 51 (3.6%) | 9 | 4 |
| AG-278-A09 | Rokumicrobia | OV-2 | 8 (2.3%) | 2 | 0 |
| ***AG-278-B04** | Rokumicrobia | OV-2 | 38 (1.5%) | 15 | 3 |
| ***AG-278-C09** | Rokumicrobia | OV-2 | 26 (1.6%) | 10 | 4 |
| ***AG-278-N11** | Rokumicrobia | OV-2 | 22 (2%) | 12 | 1 |
| ***AG-278-N20** | Rokumicrobia | OV-2 | 38 (5%) | 15 | 7 |
| ***OV-2 bin8**[3] | Infratellusbacteria | OV-2 | 64 (4.1%) | 32 | 1 |
| ***Rhizosphere bin**[3] | Rokumicrobia | Puerto Rico | 15 (0.5%) | 23 | 0 |

**Supplemental Table 5.** Summary of KO numbers for annotated genes of major energy and metabolic pathways for the Rokubacteria classes Rokumicrobia and Infratellusbacteria (also Supplemental Figure 1). X represents absent protein.

| Gene annotation | Rokumicrobia | Infratellusbacteria |
|---|---|---|
| **Glycolysis** | | |
| hexokinase | X | X |
| glucose-6-phosphate isomerase | K01810 | K01810 |
| 6-phosphofructokinase | K00850 | K00850 |
| fructose-bisphosphate aldolase | K01623 | K01623 |
| glyceraldehyde 3-phosphate dehydrogenase | K00134 | K00134 |
| phosphoglycerate kinase | K00927 | K00927 |
| 2,3-bisphosphoglycerate-independent phosphoglycerate mutase | K15633 | K15633 |
| glyceraldehyde-3-phosphate dehydrogenase | K00131 | K00131 |
| enolase | K01689 | K01689 |
| pyruvate kinase | K00873 | K00873 |
| **TCA cycle** | | |
| phosphoenolpyruvate carboxylase | K01586 | K01586 |
| citrate synthase | K01647 | K01647 |
| aconitate hydratase 1 | K01681 | K01681 |
| isocitrate dehydrogenase | K00031 | K00031 |
| 2-oxoglutarate ferredoxin oxidoreductase | K00174 | K00174 |
| succinyl-CoA synthetase | K01902 | K01902 |
| succinate dehydrogenase | K00234 | K00234 |
| fumarate hydratase A | K01676 | K01676 |
| malate dehydrogenase | K00025 | K00025 |
| **Reverse TCA** | | |
| pyruvate:ferredoxin oxidoreductase | K00169 | K00169 |
| acetyl-CoA reductase | K01895 | K01895 |
| ATP citrate lyase | X | X |
| **Wood Ljungdahl pathway** | | |
| carbon monoxide dehydrogenase | K00198 | K00198 |
| acetyl-CoA synthase | X | X |
| **DNA packaging poteins** | | |
| DNA gyrase *gyrA/B* | K02469/70 | K02469/70 |
| Topoisomerase *topA* | K03168 | K03168 |
| Single-standed DNA binding protein *ssb* | K03111 | K03111 |
| family partition protein *2 parA* + *parB* | K03496/7 | K03496/7 |
| DNA binding protein *hupB* | K03530 | K03530 |
| chromosome segregation protein *smc* | K03529 | K03529 |
| Curved DNA-binding protein *cbpA* | K05516 | X |
| Integration host factor IHF | K04764 | X |
| Starvation-inducible DNA-binding protein *dps* | K04047 | X |
| Fis (DNA binding protein) | K03557 | X |
| **Diderm membrane metabolism** | | |
| flagellar L-ring protein precursor FlgH | K02393 | X |

| | | |
|---|---|---|
| flagellar P-ring protein precursor FlgI | K02394 | X |
| Preprotein translocase SecY | K03076 | K03076 |
| Sec-independent translocation TatC protein | K03118 | K03118 |
| Prolipoprotein diacylglyceryl transferase LGT | K13292 | K13292 |
| Lipoprotein signal peptidase Peptidase_A8 | X | X |
| Bac_surface_Ag | K18827 | |
| Bacterial general secretion pathway protein D Secretin | X | X |
| Secretin_N | X | X |
| UDP-3-O-acyl-N-acetylglucosamine deacetylase LpxCD/ | K02535/6 | K02535/6 |
| Organic solvent tolerance-like, N-terminal OstA Surf_Ag_VNR | X | X |
| outer membrane lipoprotein carrier protein LolA | K03634 | K03634 |
| outer membrane lipoprotein carrier protein LolC | K09808 | K09808 |
| **Sulfur metabolism** | | |
| SOX pathway | K17222[1] | X |
| sulfane dehydrogenase subunit SoxC | X | K17225[2] |
| **Nitrogen metabolism** | | |
| ferredoxin-nitrate reductase NarB | K00367 | X |
| nitrite reductase (NO-forming) NirK | K00368 | X |
| nitrous-oxide reductase NosZ | K00376 [1] | X |
| nitrate reductase / nitrite oxidoreductase NxrA/B | K00370 | X |
| **Flagella and pili assembly** | | |
| Type IV pilus assembly protein pilC | K02653 | K02653 |
| twitching motility protein pilT | K02669 | K02669 |
| Type IV pilus assembly protein pilB | K02652 | K02652 |
| chemotaxis protein motA/B | K02556/7 | X |
| flagellar P-ring protein precursor FlgI | K02394 | X |
| flagellar L-ring protein precursor FlgH | K02393 | X |
| flagellar basal-body rod protein FlgG | K02392 | X |
| flagellin FliC | | |
| flagellar hook-associated protein 2 FliD | K02407 | X |

[1] Only annotated in one Rokumicrobia SAG from Finsch gold mine, South Africa (AD-967-E21).
[2] Only annotated in one Infratellusbacteria SAG from Crystal Spring, Nevada (AG-128-L10).

**Supplemental Table 6.** Top BLAST hits of genes from OV-2 Infratellusbacteria metagenome bin (bin 8) to Rokumicrobia single-amplified genomes (SAGs). Also see main text Figure 2.

| Gene RAST ID | | | | | |
|---|---|---|---|---|---|
| **OV-2 bin (Infratellusbacteria)** | **SAGs (Rokumicrobia)** | **% identity** | **length** | **e-value** | **SAG** |
| 215894.peg.5579 | 150852.peg.275 | 100 | 382 | 0 | AG-278-B04 |
| 215894.peg.5309 | 150853.peg.21 | 99.88 | 801 | 0 | AG-278-C09 |
| 215894.peg.2670 | 140332.peg.919 | 94.89 | 333 | 0 | AD-967-E21 |
| 215894.peg.664 | 132753.peg.570 | 93.15 | 788 | 0 | AG-159-M09 |
| 215894.peg.2669 | 150857.peg.933 | 91.24 | 411 | 0 | AG-278-N11 |
| 215894.peg.4356 | 132754.peg.281 | 86.51 | 415 | 0 | AG-159-N17 |
| 215894.peg.1331 | 132749.peg.48 | 75.78 | 574 | 0 | AG-159-B15 |
| 215894.peg.1090 | 140334.peg.57 | 74.88 | 633 | 0 | AG-138-D06 |

**Supplemental Table 7.** JGI IMG accession numbers for all Rokubacteria single amplified genomes and metagenomes.

| **SAG name** | **IMG ID** |
|---|---|
| AG-159-B15 | 2651869898 |
| AG-160-L13 | 2651869893 |
| AG-159-M09 | 2651869895 |
| AG-159-L22 | 2651869896 |
| AG-278-N11 | 2651869891 |
| AG-159-P01 | 2713897487 |
| AG-160-P19 | 2651869612 |
| AG-278-B04 | 2651869612 |
| AG-138-D06 | 2651869899 |
| AG-159-G23 | 2651869897 |
| AG-128-L10 | 2651869900 |
| AD-967-E21 | 2651869901 |
| AG-278-C09 | 2651869892 |
| AG-159-N17 | 2651869894 |
| AG-657-I10 | 2747842444 |
| AG-657-L06 | 2747842445 |
| AG-657-A01 | 2747842443 |
| AG-278-A09 | 2747842441 |
| AG-278-N20 | 2747842442 |
| | |
| **Metagenome** | |
| Oasis Valley 2 (P3) | 330009444 |
| Oasis Valley 2 (P2) | 330009691 |
| Oasis Valley 2 (P1) | 330009626 |
| Tabelua rhizophere | 330005985 |

## References


Anantharaman, K., Brown, C.T., Hug, L.A., Sharon, I., Castelle, C.J., Probst, A.J. et al. (2016) Thousands of microbial genomes shed light on interconnected biogeochemical processes in an aquifer system. *Nat Commun* **7**: 13219.

Butterfield, C.N., Li, Z., Andeer, P.F., Spaulding, S., Thomas, B.C., Singh, A. et al. (2016) Proteogenomic analyses indicate bacterial methylotrophy and archaeal heterotrophy are prevalent below the grass root zone. *PeerJ* **4**: e2687.

Dillon, S.C., and Dorman, C.J. (2010) Bacterial nucleoid-associated proteins, nucleoid structure and gene expression. *Nat Rev Microbiol* **8**: 185-195.

Hartman, T., Weinrick, B., Vilcheze, C., Berney, M., Tufariello, J., Cook, G.M., and Jacobs, W.R., Jr. (2014) Succinate dehydrogenase is the regulator of respiration in Mycobacterium tuberculosis. *PLoS Pathog* **10**: e1004510.

Hug, L.A., Thomas, B.C., Sharon, I., Brown, C.T., Sharma, R., Hettich, R.L. et al. (2016) Critical biogeochemical functions in the subsurface are associated with bacteria from new phyla and little studied lineages. *Environ Microbiol* **18**: 159-173.

Hugler, M., Wirsen, C.O., Fuchs, G., Taylor, C.D., and Sievert, S.M. (2005) Evidence for autotrophic CO2 fixation via the reductive tricarboxylic acid cycle by members of the epsilon subdivision of proteobacteria. *J Bacteriol* **187**: 3020-3027.

Lee, S.Y., Lim, C.J., Droge, P., and Yan, J. (2015) Regulation of Bacterial DNA Packaging in Early Stationary Phase by Competitive DNA Binding of Dps and IHF. *Sci Rep* **5**: 18146.

Parks, D.H., Imelfort, M., Skennerton, C.T., Hugenholtz, P., and Tyson, G.W. (2015) CheckM: assessing the quality of microbial genomes recovered from isolates, single cells, and metagenomes. *Genome Res* **25**: 1043-1055.

Sorokin, D.Y., Lucker, S., Vejmelkova, D., Kostrikina, N.A., Kleerebezem, R., Rijpstra, W.I. et al. (2012) Nitrification expanded: discovery, physiology and genomics of a nitrite-oxidizing bacterium from the phylum Chloroflexi. *ISME J* **6**: 2245-2256.
